# Consensus, controversies, and conundrums of P4-ATPases: The emerging face of eukaryotic lipid flippases

**DOI:** 10.1016/j.jbc.2024.107387

**Published:** 2024-05-17

**Authors:** H. Diessel Duan, Huilin Li

**Affiliations:** Department of Structural Biology, Van Andel Institute, Grand Rapids, Michigan, USA

**Keywords:** ATPase, autophosphorylation, conformational change, membrane trafficking, lipid transport, inhibition mechanism, flippase, P-type ATPase, membrane asymmetry, membrane curvature

## Abstract

The cryo-EM resolution revolution has heralded a new era in our understanding of eukaryotic lipid flippases with a rapidly growing number of high-resolution structures. Flippases belong to the P4 family of ATPases (type IV P-type ATPases) that largely follow the reaction cycle proposed for the more extensively studied cation-transporting P-type ATPases. However, unlike the canonical P-type ATPases, no flippase cargos are transported in the phosphorylation half-reaction. Instead of being released into the intracellular or extracellular milieu, lipid cargos are transported to their destination at the inner leaflet of the membrane. Recent flippase structures have revealed multiple conformational states during the lipid transport cycle. Nonetheless, critical conformational states capturing the lipid cargo “in transit” are still missing. In this review, we highlight the amazing structural advances of these lipid transporters, discuss various perspectives on catalytic and regulatory mechanisms in the literature, and shed light on future directions in further deciphering the detailed molecular mechanisms of lipid flipping.

The year 2023 marks the 100th anniversary of the nomenclature “lipid” coined by the French biochemist Gabriel Bertrand. These amphipathic molecules (Fig. 1, *A* and *B*) could self-assemble into an approximately 4 nm thick bilayer film in aqueous solution. The lipid bilayer is also referred to as the membrane in biology that defines the boundary between life and nonlife or between self and nonself. The bilayer architecture of the membrane imposes strong restrictions on the movement of component lipid molecules: Lipids can move readily and rapidly within a leaflet *via* lateral diffusion but cannot flip or flop between the two leaflets due to the membrane hydrophobic core that prevents their transverse diffusion. In this review, we refer to the cytosol-facing leaflet as the inner leaflet and the leaflet facing away from cytosol as the outer leaflet, regardless of their cellular origins.

**Figure 1:**
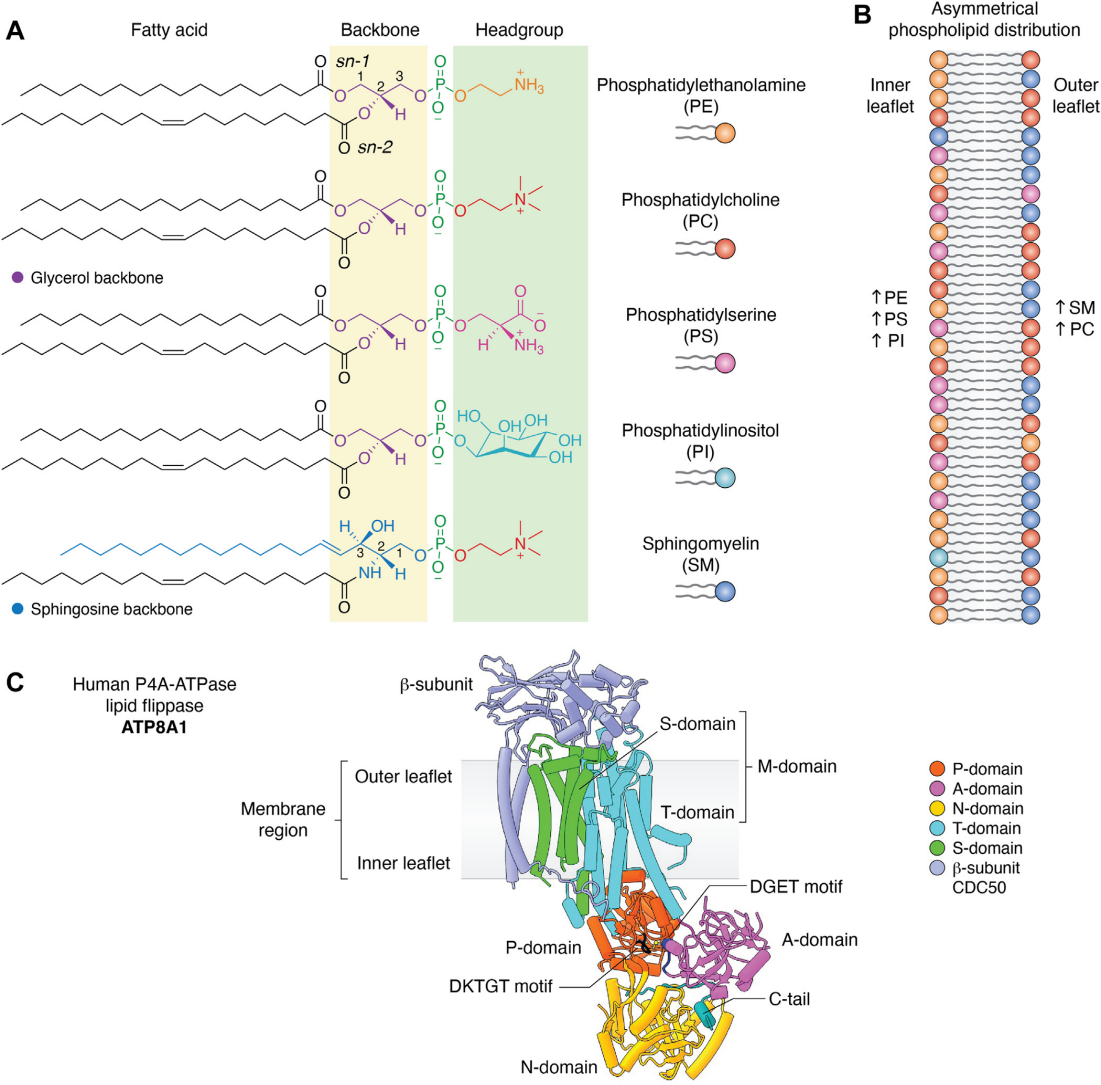
**Major phospholipids, membrane asymmetry, and structure of the human P4A-ATPase lipid flippase ATP8A1.***A*, chemical structures of phospholipids with the headgroup and backbone highlighted. The backbone carbon atoms are numbered for PE and SM to distinguish the two acyl chains (*sn-1 versus sn-2*). *B*, data-driven (84) schematic diagram of the asymmetrical distribution of phospholipids in plasma membrane of human erythrocytes. *C*, cartoon representation of human ATP8A1 (PDB 6K7L) with the DKTGT motif of the P-domain for phosphorylation highlighted in black and the DGET motif of the A-domain for dephosphorylation highlighted in *dark blue*.

Different types of lipid molecules in eukaryotes are uniformly distributed between the two leaflets when they are first synthesized at the endoplasmic reticulum (ER) membrane (1). However, their distribution becomes asymmetric in the Golgi, endosomal, and plasma membranes, a phenomenon called membrane asymmetry. The inner leaflet becomes enriched with lipids such as phosphatidylethanolamine (PE), which bears a small headgroup, and phosphatidylserine (PS), which carries a negative charge; meanwhile, the outer leaflet is enriched with neutral lipids such as sphingomyelin (SM) and, to a lesser extent, phosphatidylcholine (PC) (Fig. 1, *A* and *B*) (2, 3). On a cellular level, regulation of such compositional asymmetry is fundamental to many cellular processes such as membrane trafficking, cell polarization, blood coagulation, myoblast fusion, and apoptosis (4, 5). On an organismal level such as mammals, the dysregulation is implicated in pathophysiological conditions such as intrahepatic cholestasis (6, 7), insulin resistance (8, 9), obesity (10, 11), sperm capacitation anomalies (12, 13), progressive hearing loss (14, 15), Alzheimer disease (16, 17), and tumorigenesis (18, 19, 20). The enrichment of PS and PE at the inner leaflet is achieved by the P4-ATPase lipid flippases that harness the energy of ATP hydrolysis to unidirectionally flip these lipids from the outer leaflet to the inner leaflet. The letter P describing the ATPase type refers to autophosphorylation of an invariant aspartate residue by ATP at the catalytic site. Although both ATP and phospholipids are considered substrates for P4-ATPases in the literature, phospholipids may be more accurately described as “cargos” (21, 22), given that the lipid molecules do not undergo chemical transformation. The ATP hydrolysis assay is often used as a facile proxy to assess the enzymatic activity of purified flippase proteins, taking advantage of the enzyme’s coupling of ATP hydrolysis with cargo transport, but a flippase assay by reconstitution in liposomes and use of analogs such as fluorophore-labeled lipids is still essential to probe the flippases’ cargo preference and activity (23).

The ATP-dependent transport of PS and PE to the inner leaflet was first observed in the human erythrocyte membrane using spin-labeled lipid analogs, implying the presence of a lipid transporter with the energy input from ATP (24). Such a proposed lipid transporter—the first lipid flippase ATPase II (a P4-ATPase later renamed as ATP8A1)—was purified from bovine adrenal glands and found to be homologous to the yeast Drs2 (25). Over 2 decades after the initial discoveries, the first complete structures of the human ATP8A1 and the yeast Drs2 were determined by cryo-EM in 2019 and their mechanisms of transport cycle (26), autoinhibition, and activation (27, 28) were proposed. These were followed in the ensuing years by structures (Table 1) of thermophilic fungus *Chaetomium thermophilum* Dnf1 in nanodiscs (29), human ATP11C in detergents (30, 31), yeast Dnf1 and Dnf2 in detergents (32), additional states of yeast Drs2 (33), yeast Neo1 (34), human ATP11C in nanodiscs (35), yeast Dnf1 in both detergents and nanodiscs (36), and human ATP8B1 (37, 38, 39). P4-ATPases are recently proposed to be divided phylogenetically into three subfamilies of P4A, P4B, and P4C, with P4C being an intermediate group between P4A and P4B (40). Most flippases contain a noncatalytic β-subunit which belongs to the Cdc50 family of proteins and functions in protein export at the ER (41) and lipid cargo loading (42). However, the yeast Neo1, a P4B subfamily member, does not have a β-subunit.

**Table 1:** Reported flippase structures with individual PDB identification code in various conformational states

Flippases	Conformational states						
	E1	E1·ATP	E1∼P·ADP	E1∼P	E2P	E2∼P	Orphan
ATP8A1	6K7G 6K7H	6K7I 6K7J	6K7K	6K7N	6K7L	6K7M	
ATP8B1		8OX4^a^	8OX5^a^	8OX6^a^^,^^b^	7PY4 7VGH 7VGI 8OX7 8OX8 8OX9	7VGJ^c^ 8OXA 8OXB 8OXC	
ATP11C		7BSP	7BSQ	7VSH^d^ 7BSS^b^	6LKN 7BSU^b^	7BSV^b^ 7BSW^b^ 7VSG^b^^,^^d^	
Dnf1	7KY6^a^	7WHW 7DSH^d^	7KYB^a^		7KYC 7WHV 7DRX^d^		7DSI^d^ 7F7F^d^
Dnf2	7KY7^a^	7KY8^a^	7KY9^a^		7KYA	7KY5^e^	
Drs2	6PSX 7OH4	7OH7	7OH5		6PSY 6ROH 6ROI 6ROJ 7PEM	7OH6	
Neo1		7RD8			7RD6	7RD7^e^	
Dnf1 from *Chaetomium thermophilum*		6LCR^d^			6LCP^d^		

a A-domain unresolved.

b N-domain unresolved.

c AlF_4_^-^unresolved.

d In nanodiscs.

e Lipid occlusion unobserved.

## Old scaffold, new function: General themes of lipid flippases

P4-ATPase flippases are built on the same scaffold as the canonical cation-transporting P2-ATPases such as Na^+^/K^+^-ATPases and Ca^2+^-ATPases (Fig. 1*C*). The scaffold contains a ten-helix transmembrane domain (M-domain) and three cytoplasmic domains involved in the reaction cycle of phosphorylation and dephosphorylation of an invariant aspartate: the nucleotide binding (N)-domain, phosphorylation (P)-domain, and actuator (A)-domain (43, 44, 45, 46, 47). The N-domain binds ATP, the P-domain contains the conserved DKTGT motif that provides the aspartate for initial phosphorylation, and the A-domain bears the DGET motif (TGES motif in P2-ATPases) that provides a conserved glutamate to facilitate dephosphorylation of the aspartyl phosphate intermediate (48, 49). The 10 transmembrane helices are historically further divided into a transport (T)-domain (M1-M6) for cargo binding and transport and a structural support (S)-domain (M7-M10) (50, 51, 52, 53, 54). The heterodimeric P4A-ATPase flippases are the most ubiquitous in all eukaryotes with an indispensable β-subunit that belongs to the Cdc50 family of proteins. Cdc50 contains two transmembrane helices connected to a large ectodomain that is stabilized by multiple N-glycans and two disulfide bonds (41, 55). It is currently unclear whether the single-subunit P4B flippases evolved first and other types evolved later by gaining the β-subunit, or the other way around, in which case the P4B flippases evolved later from the two-subunit flippases by losing the β-subunit (34, 40).

**Figure 2:**
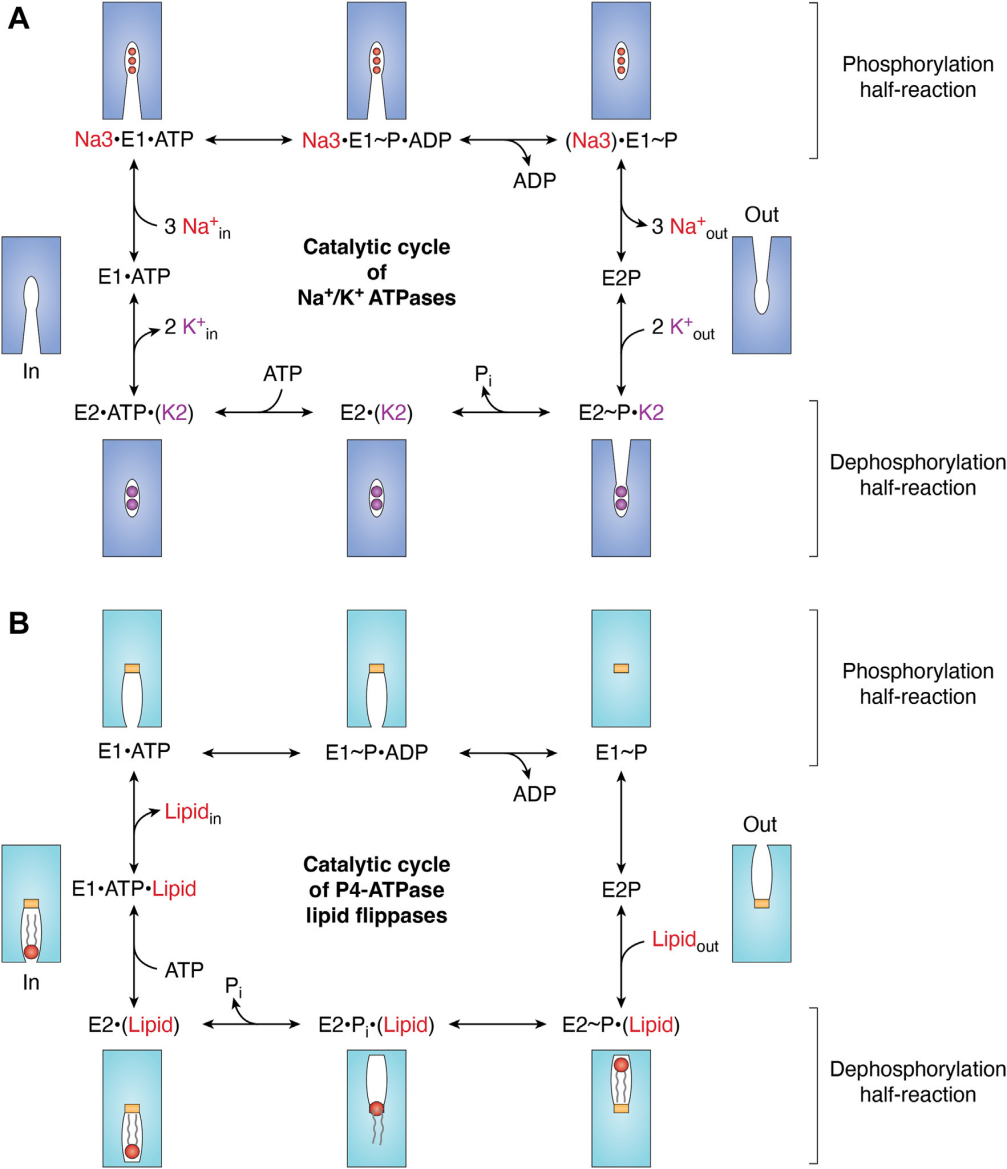
**The catalytic reaction schemes of Na**^**+**^**/K**^**+**^**-ATPases (*A*) and P4-ATPase lipid flippases (*B*) based on the E1/E2 model.** Starting from the state at the top-left corner clockwise, the reaction cycle can be divided into the phosphorylation half-reaction (*top row*) and dephosphorylation half-reaction (*bottom row*), with the facing orientation changing into the outward-facing (*right column*) and the inward-facing (*left column*). Substrate, product, and cargo-bound states are depicted by a middle dot, cargo-occluded states are placed in parentheses, and transition states are indicated by a tilde. Na3 and K2 denote the three protein-bound Na^+^ and the two protein-bound K^+^, respectively. E1 and E2 indicate the phosphorylation phase and dephosphorylation phase, respectively. The small gold band in the middle of flippases represents a hydrophobic barrier that the lipid head has to cross.

The E1/E2 scheme or the Post-Albers scheme further developed by de Meis and later by Glynn has been widely adopted to depict the reaction cycle for P-type ATPases, in particular for the cation-transporting P2-ATPases (Fig. 2*A*) (56, 57, 58, 59). The lipid transport cycle of P4-ATPase flippases has been portrayed largely following that of P2-ATPases (43, 44, 60, 61), however, flippases are different from P2-ATPases in that they have no transport activity in the first half-reaction, except for a priming event of autophosphorylation by ATP. The second half-reaction of flippases is the actual lipid transport which is coupled with the dephosphorylation event. Nonetheless, unlike P2-ATPases whose transported cations are released into the cytosol or luminal and extracellular space, the hydrophilic head of lipid cargos of flippases travels in the transport pathway until it reaches the destination at the inner leaflet of the membrane (Fig. 2).

For flippases, the transport-silent E1 phase prepares the protein for the subsequent lipid cargo transport during the E2 phase, through ATP binding (E1·ATP), phosphoryl transfer from ATP to aspartate (E1∼P·ADP), and phosphorylated high-energy intermediate (E1∼P), until transition to the phosphorylated ground-state intermediate (E2P) (Fig. 2*B*). It is generally assumed that the E1 phase is an inward-facing conformation, *that is*, the cargo exit site is open toward the membrane inner leaflet, while the E2 phase is an outward-facing conformation, *i.e.*, the cargo entry site is open toward the membrane outer leaflet. This assumption is consistent with the fact that all reported flippase E1 phase structures (including E1, E1·ATP, E1∼P·ADP and E1∼P) harbor a closed entry to the lipid binding site. Transition to the low-energy E2P state opens up a hydrophilic cleft toward the outer leaflet to initiate lipid cargo loading and binding of the lipid polar headgroup. However, this mechanism is not universal because an open cleft has not been observed in the E2P state of ATP8A1 (26), the “closed” autoinhibited ATP8B1 (38, 39) and the autoinhibited Drs2 (27, 28). Therefore, there appears to be an equilibrium between the “open” and “closed” conformations at the lipid entry site in the E2P state of flippases. Interestingly, a lipid-occluded E2∼P state conformation was observed in nanodisc-reconstituted ATP11C, instead of the intended E2P state (35). A phosphate analog, BeF_3_^−^, was used in that work to capture the E2P state, but the high PS concentration inside the nanodisc may have driven the transport process one step further than expected, *that is*, from the lipid-entry-open E2P state to the lipid-entry-closed E2∼P state with an occluded PS (35). Structures of states following the lipid-occluded E2∼P state are currently unknown. Therefore, it is largely unclear how and when the lipid cargo is released from the transport pathway to the inner leaflet of the membrane. Nonetheless, lipids in a putative cargo exit site have been observed in the fungal Dnf1 flippases in their respective E2P states (29, 32, 36). These lipids are wedged between transmembrane helices 2 and 4 (M2 and M4) in the inner leaflet of the membrane and are presumed to be the cargo lipids carried over from a previous transport cycle, but the possibility of them being annular lipids cannot be ruled out.

## Transport-silent priming phase: What exactly is going on?

**Figure 3:**
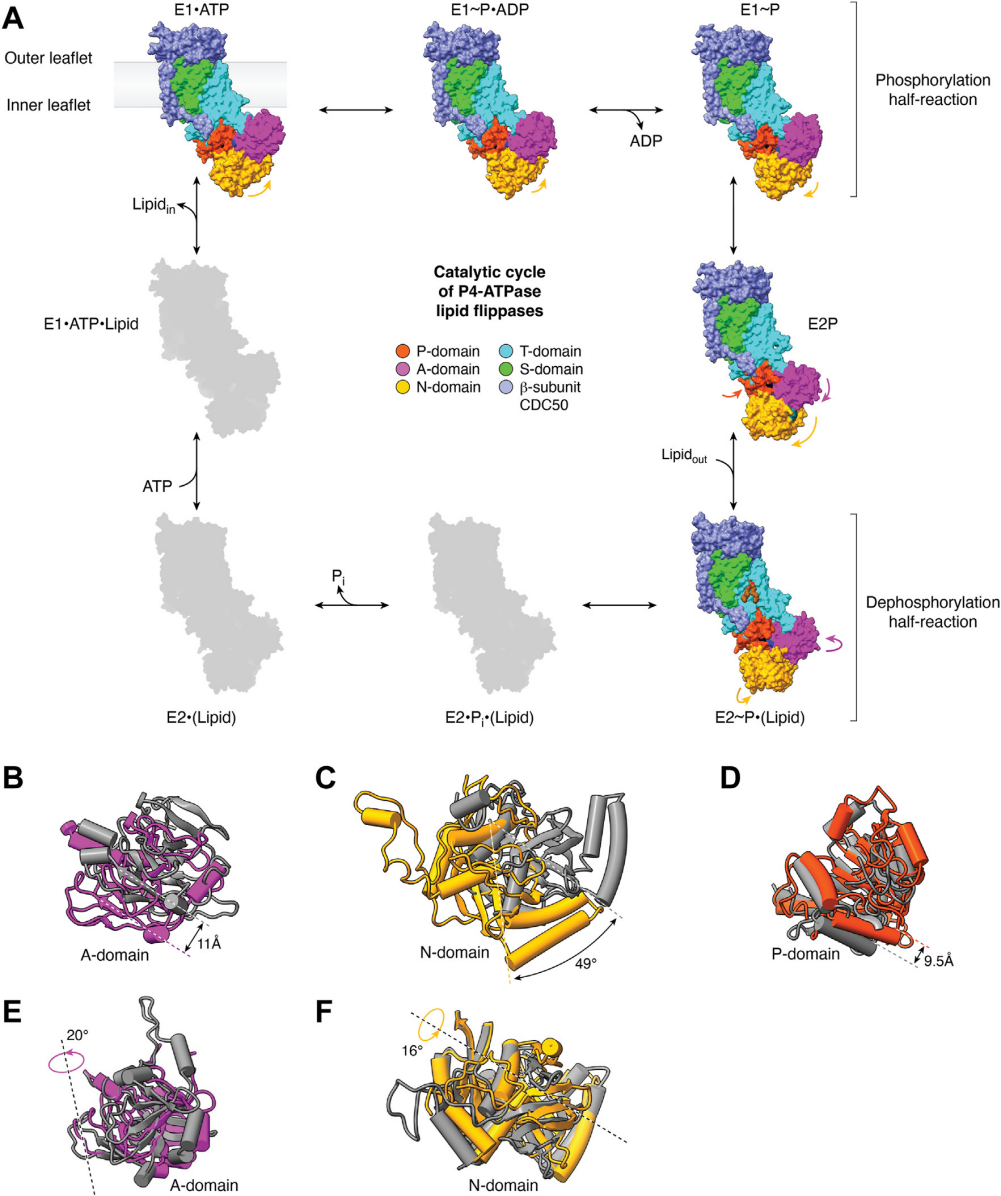
**Cytosolic A, P, and N domains undergo large-scale conformational changes during the catalytic cycle of P4-ATPase lipid flippases (*A*) especially during lipid loading (*B*, *C* and *D*) and occlusion (*E* and *F*).** The states are based on the E1/E2 model. Starting from the *top-left* ATP-bound state and going clockwise, in the phosphorylation half-reaction (*top row*), N-domain rotates (E1·ATP, PDB 6K7J) until fully associated with P-domain and then transfers the γ-phosphate from ATP to the catalytic aspartate in P-domain (E1∼P·ADP, PDB 6K7K). N-domain releases ADP and rotates away from P-domain (E1∼P, PDB 6K7N) to complete the phosphorylation. Concurrent rotations of both N-domain and A-domain allow N-domain to be fully dissociated with P-domain and open the lipid entry *via* M1 and M2 that are connected to A-domain (E2P, PDB 6K7L). A small inclination of P-domain allows its connected M4 to accommodate the lipid binding as well (E2P). In the dephosphorylation half-reaction (*bottom row*), A-domain rotates horizontally to fully associate with P-domain and reorients its catalytic glutamate to remove the aspartyl phosphate in P-domain (E2∼P·(Lipid), PDB 6K7M). This in turn closes the lipid entry *via* A-domain connected M1 and M2 and occludes the lipid cargo (E2∼P·(Lipid)). Meanwhile, the N-domain also tilts toward A-domain to promote A-domain rotation (E2∼P·(Lipid)). The lipid molecule is shown as brown spheres. Remaining conformational states are shown in *gray* to indicate the lack of structural information. For the state names, substrate, product, and cargo-bound states are depicted by a middle dot, cargo-occluded states are placed in parentheses, and transition states are indicated by a tilde. *B*, movement of A-domain from the E1∼P state (PDB 7VSH, gray) to the E2P state (PDB 6LKN). *C*, concurrent rotation of N-domain from the E1∼P state (PDB 7VSH, gray) to the E2P state (PDB 6LKN). *D,* movement of P-domain from the E1∼P state (PDB 7VSH, gray) to the E2P state (PDB 6LKN). *E*, rotation of A-domain from the E2P state (PDB 8OX9, gray) to the E2∼P·(Lipid) state (PDB 8OXB). *F*, concurrent rotation of N-domain from the E2P state (PDB 8OX9, gray) to the E2∼P·(Lipid) state (PDB 8OXB). The conformational dynamics were analyzed by DynDom (85).

One profound distinction that sets P4-ATPase flippases apart from canonical P2-ATPases is the lack of cargo transport during the autophosphorylation half-reaction (48, 62, 63), which is otherwise triggered by the binding of Na^+^ or Ca^2+^ ions for Na^+^/K^+^-ATPases and Ca^2+^-ATPases, respectively. In the absence of any ligands, the A and N domains are highly mobile in the inward-facing E1 state. ATP8A1 exists in two conformations with an RMSD of 3.1 Å in the E1 state, due to the mobile A and N domains (26). The A-domain is fully mobile in the E1 state of Dnf1 and Dnf2 and becomes invisible in averaged 2D EM images (32). ATP binding brings the N-domain closer to the P-domain for the following phosphoryl transfer from ATP to the invariant aspartate (Fig. 3*A*). Nonetheless, the N-domain remains largely mobile such that the distance between the ATP’s γ-phosphate moiety and the catalytic aspartate can vary from below 3.5 Å in a near attack conformation (NAC) (29, 31, 36, 39) to 5∼7 Å when the N-domain is only partially engaged with the P-domain (26, 32, 33, 34, 36). The A-domain seems to become even more mobile in the E1·ATP state than in the E1 state. While the A-domain of Dnf2 remains invisible in the E1·ATP state as in the E1 state (32), the flippase-specific DGET motif carrying the invariant glutamate from the A-domain of ATP8A1 is over 12 Å away from the catalytic aspartate in the P-domain (26), and the distance is even larger at 24 Å in the *C. thermophilum* Dnf1 reconstituted in nanodiscs (29). The large distance places the A-domain well into the membrane core if the membrane adjacent to the flippase remains flat. Because ATP binding in Na^+^/K^+^-ATPases accelerates the rate-limiting E2 to E1 transition, leading to the release of K^+^ to the cytosol in the inward-facing conformation (64, 65), one could envision that the mobile flippase A-domain may facilitate lipid cargo release through its connected transmembrane helix 2 (M2) in a similar ATP-dependent manner (Fig. 2*B*).

The subsequent phosphoryl transfer through nucleophilic attack by the hydroxyl oxygen from the P-domain catalytic aspartate generates phosphorylated aspartate, with ADP as the leaving group. For flippases whose N-domain is already fully associated with the P-domain in the E1·ATP state, ATP hydrolysis leads to the E1∼P·ADP state with little conformational change, as the structures are largely superimposable to that of E1·ATP (31, 39). Otherwise, the N-domain rotates further toward the P-domain to phosphorylate the P-domain catalytic aspartate (Fig. 3*A*) (26, 32, 33). Upon release of ADP, the N-domain becomes highly mobile in the E1∼P state, presumably due to loss of the bridging ligand. Indeed, the N-domain becomes invisible in the E1∼P state of ATP11C in detergents (31); the N-domain density is poorly defined in the E1∼P state of ATP8B1 (39). The N-domain rotates away and dissociates from the P-domain in the E1∼P state of ATP8A1 and nanodisc-reconstituted ATP11C, leaving behind the P-domain now bearing a phosphorylated high-energy intermediate (Fig. 3*A*) (26, 35). Such dissociation prevents the phosphoryl group from reassociating with ADP and forming ATP again, because the E1∼P state is ADP-sensitive (48, 66, 67, 68). This is in contrast to the ground-state E2P in the dephosphorylation half-reaction in which the flippases are cargo-sensitive but become ADP-insensitive. Taken together, the lack of structural changes in the cargo-transporting transmembrane region in the E1 phase supports the notion that flippase phosphorylation is independent of a transported cargo and primes the enzyme for the lipid transport in the second half-reaction, *that is*, the E2 phase.

## E2P under scrutiny: Is it autoinhibition or autoregulation?

The transition from E1∼P to E2P has been visualized for ATP8A1 and ATP11C flippases. These structures consistently reveal that the N-domain fully dissociates from the P-domain in the ADP-insensitive E2P state, accompanied by the A-domain DGET motif associating with the P-domain for the subsequent dephosphorylation (26, 30, 35). A slight inclination of the P-domain toward the A-domain is also clear in both flippases (Fig. 3, *A* and *D*). In ATP11C, the N-domain rotates 49° to make room for the A-domain to move in and bind to the P-domain (Fig. 3*C*). The A-domain movement (Fig. 3*B*) rearranges the connected transmembrane helices M1 and M2, leading to the opening of the lipid entry site at the outer leaflet. Meanwhile, the above-mentioned P-domain inclination reorients the so-called “hydrophobic gate” residue Val-357 at the kink position of the connected M4 to accommodate the lipid headgroup (53).

**Figure 4:**
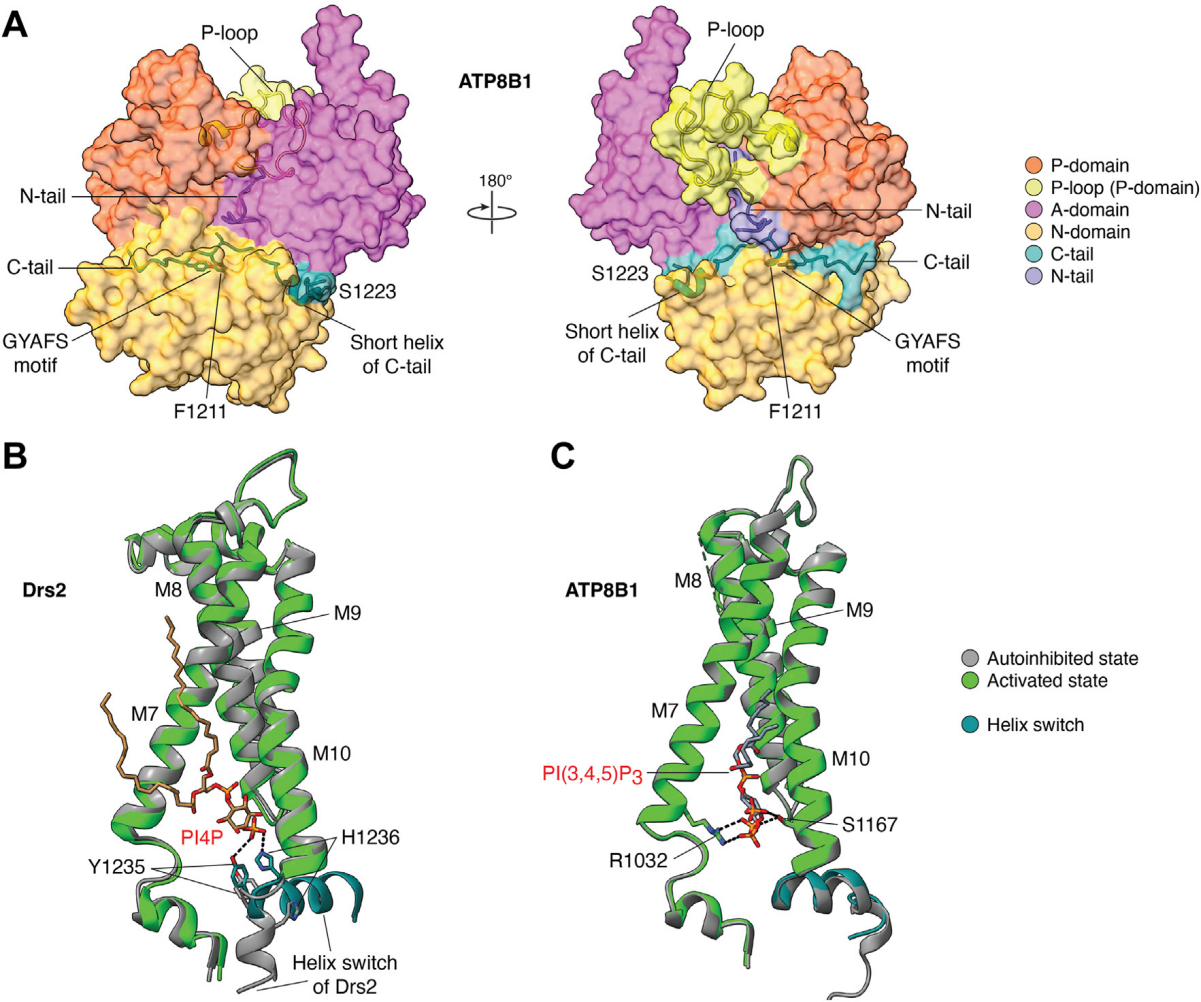
**Regulation of the human ATP8B1 flippase activity by the C-tail, N-tail, and P-loop of the P-domain (*A*) and phosphoinositide binding in Drs2 and ATP8B1 (*B* and *C*).***A*, transparent surface representation of the cytosolic domains of human ATP8B1 (PDB 7VGI) with key residues of the C-tail shown in sticks. *B*, Drs2 in the autoinhibited state (PDB 6PSY) superimposed with the activated state (PDB 6ROJ). Key residues of the “helix switch” in the activated Drs2 interacting with PI4P are shown in sticks. PI4P is shown as brown sticks. *C*, ATP8B1 in the autoinhibited state (PDB 8OX8) superimposed with the activated state (PDB 8OX9). The equivalent “helix switch” in the activated ATP8B1 is also shown for comparison. Key residues interacting with PI(3,4,5)P_3_ are shown in sticks. For clarity only the PI(3,4,5)P_3_ in the activated ATP8B1 is shown as *slategray* sticks.

The E2P state can be formed either by autophosphorylation or by incubation with the phosphate analog BeF_3_^−^ (69). A signature structural element often associated with the E2P state is a C-terminal extension (termed “C-tail” hereafter) bearing a GYAFS motif plus a seven-residue short α-helix (Fig. 4*A*) (26, 27, 28). The C-tail threads horizontally through the interface between N and P domains as well as the interface between N and A domains and appears to tie them together, thus stabilizing these domains. The C-tail is not strictly conserved in flippases. For example, P4B-ATPases do not possess such a C-tail. Some flippases with a C-tail may lack the GYAFS motif, making the C-tail disordered in the E2P state, as has been observed in the yeast Dnf1 and Dnf2 and the human ATP11C (31, 32). The functional importance of the C-tail was demonstrated by a progressive truncation of the C-tail of the bovine ATP8A2. Deleting the seven-residue short helix alone compromised the PS-stimulated ATP hydrolysis activity as well as lipid flipping activity, but deleting both the short helix and GYAFS motif only slightly further decreased these activities. Interestingly, deleting the entire C-tail restored both ATPase and flippase activities (70). These observations suggest that the C-tail regulates rather than simply autoinhibits the ATP8A2 activity. Indeed, the ATP8A1 C-tail short helix was proposed to keep the N-domain away from the A-domain in the E2P state, thereby allowing the A-domain to move and open the lipid entry site. The invariant phenylalanine in the GYAFS motif was proposed to occupy the adenine moiety binding site and prevent ATP binding at the N-domain (Fig. 4*A*) (26). However, the flippase structures in the E2P state are essentially superimposable with or without the ordered C-tail. Therefore, it remains to be investigated why removing the C-tail can restore the enzyme activities.

A better-understood role of the C-tail is that it can keep flippases in an autoinhibited state (27, 28, 37, 38, 39). Due to this C-tail mediated autoinhibition, purified endogenous yeast Drs2 was inactive. Nonetheless, the enzyme could be activated *in vitro* by incubation with the phosphoinositide PI4P (phosphatidylinositol 4-phosphate) (28). Interestingly, the autoinhibited Drs2 is in the E2P state, with an unmodeled EM density of the size of a phosphate adjacent to the invariant Asp-560, suggesting that the flippase rests in an autophosphorylated state (28). Interestingly, the same resting state of autophosphorylated E2P structure was observed in an overexpressed human ATP8B1 flippase as well (37). Because of the high cellular ATP concentration and the transport-silent nature of the phosphorylation (E1) phase, it is tempting to speculate that these flippases rest in an autophosphorylated E2P state, ready to bind a lipid cargo for a dephosphorylation-coupled lipid transport process. However, the available flippase structures in the autophosphorylated E2P state conflict with the proposed C-tail role in preventing ATP binding through its GYAFS motif, because phosphorylation has already occurred, presumably from ATP, and blocking ATP binding does not appear to be necessary at this stage. Indeed, the autoinhibited ATP8B1 was able to be phosphorylated with ATP (38, 39). This conundrum has led to the hypothesis that the C-tail is in equilibrium between bound and unbound forms, and the equilibrium shifts toward the bound form in the E2P state (38). Alternatively, the C-tail GYAFS motif may prevent ADP rather than ATP binding, explaining why flippases are not sensitive to ADP in the E2P state. Regardless, the C-tail strings all three cytosolic domains (A, N and P domains) together such that the conformational changes required for catalysis are no longer available (Fig. 4*A*).

## Flippase activation: “Simple but difficult” *versus* “Complex but easy”?

For flippases that have a C-tail with both the signature GYAFS motif and short α-helix, it remains enigmatic why some (*e.g.*, Drs2 and ATP8B1) require activation (27, 28, 37, 38, 39), while others (*e.g.*, ATP8A1) do not (26). An autoinhibited flippase is in an off-cycle inactive E2P state and must be activated for function. The yeast Drs2 can be activated *in vitro* either by removing the 121-residue C-tail or by PI4P, and removal of the 121-residue C-tail negates the requirement for PI4P for activation (71). These observations suggest that PI4P activates Drs2 by interaction with the C-tail. Comparing the structures of autoinhibited and activated full-length Drs2 revealed that PI4P relieves the inhibition by dislodging the C-tail from the extended binding groove thereby liberating the otherwise restrained A, N, and P domains. Accompanying such activation is the transformation of Drs2 from the inactive E2P state to an active E1 state. Interestingly, no PI4P was observed in the activated E1 structure, suggesting that PI4P has diffused away post activation and PI4P binding is not required to sustain the activated state (28). This scenario was rationalized by the presence of a “helix switch” in the C-tail carrying two key residues Y1235 and H1236 for PI4P binding (Fig. 4*B*); both residues were shown to be essential for ATPase activity, presumably due to the fact that their mutations had disrupted PI4P binding for activation. This mechanism also explains the observed PI4P-independence for the 121-residue (ΔT1234-I1355) C-tail cleaved Drs2, as the resultant protein also lost PI4P binding in addition to its C-tail.

Activation of Drs2 can also be accomplished by truncation of the C-tail which is a clear-cut approach albeit less physiologically relevant (27). Interestingly, upon activation, Drs2 with a partially truncated C-tail (ΔS1247-I1355) that still retains the “helix switch” captured a bound PI4P (Fig. 4*B*). This observation corroborates the proposed binding site for PI4P as an activator for Drs2 and suggests that PI4P is still required for activation provided that the “helix switch” is present. It is likely that the E2P state mimicked by BeF_3_^−^ in this case stabilized PI4P binding, while PI4P does not bind Drs2 in the E1 state. Interestingly, an opened lipid entry site is observed only in the activated and C-tail cleaved Drs2 in the E2P state but not in the autoinhibited or partially activated structures (27). It is unclear why PI4P could not fully activate Drs2 without C-tail truncation in this case (27). One possibility is that the *in vitro* PI4P activation is a rate-limiting process (28); another possibility is that trapping Drs2 in the E2P state by BeF_3_^−^ had prevented its activation by PI4P from being judged, presumably due to the resemblance between the two E2P structures for full-length proteins. This in turn begs the question: Is the C-tail of full-length Drs2 ordered as in ATP8A1 or disordered for an active E2P state during the catalytic cycle? Potentially this could be addressed by a structure of full-length Drs2 that is first activated by PI4P and then trapped in the E2P state by incubation with BeF_3_^−^. Further investigation is needed to understand the C-tail function and behavior for Drs2.

Autoinhibition is not limited to the yeast flippase Drs2. The human ATP8B1 is also autoinhibited in the as-purified form, but the detailed inhibition mechanism is slightly different. The ATP8B1 contains an N-terminal extension (termed “N-tail” hereafter) that also contributes to the inhibition. The N-tail inserts between the A and P domains and further extends to the interface of the N and P domains (Fig. 4*A*) (37, 38). Interestingly, the N-tail becomes disordered in a C-tail truncated active ATP8B1 in the E2P state, suggesting the N-tail only reinforces its inhibition in the presence of the C-tail and contributes to C-tail-mediated inhibition by cementing the cytosolic domains and hindering their conformational changes (39). Two conformations were found to co-exist in the autoinhibited ATP8B1 in the E2P state (39). These two conformations are overall similar with an RMSD of 1.3 Å except at the M1 and M2, which move to open up the lipid entry groove and reorient the “hydrophobic gate” residue Ile-402 (equivalent to Val-357 in ATP11C as discussed above) at the kink position of M4. It is likely that the M1, M2, and the kinked loop at M4 are inherently mobile, because both the A and P domains connecting to these transmembrane helices remain stationary between the open and closed conformations and could not have caused these movements.

The activation mechanism of the human ATP8B1 is also controversial. Autoinhibition of the ATP8B1 isolated from mammalian cells can be readily relieved by a cargo lipid PS (37). PS is the most favored cargo of ATP8B1 that stimulates the ATP hydrolysis activity of the flippase in both detergents and proteoliposomes and demonstrates appreciable flippase activity as well. In contrast, PC and PE do not stimulate the ATPase activity, and there is also no discernible flippase activity with PC (37). Therefore, the apparent autoinhibition may be an in-cycle state of E2P during the catalytic cycle of ATP8B1. Bile acids are naturally present in the cytosol of hepatocytes whose membranes harbor ATP8B1. Given that ATP8B1 is associated with intrahepatic cholestatic disorders (6), the effect of bile acids was also investigated. Interestingly, it was found that bile acids synergistically stimulated the ATPase activity of ATP8B1 in the presence of PS, likely by binding to a highly positively charged loop named the P-loop in the P-domain (37). The P-loop is conserved only among ATP8B1 orthologs and is proposed as a putative binding site for bile acids owing to the anionic character of these acids. The P-loop is adjacent to the autoinhibitory N-tail (Fig. 4*A*); bile acid binding at the P-loop may facilitate the release of the N-tail thereby enhancing the ATPase activity (37). This hypothesis is supported by the observation that the P-loop also becomes disordered together with the N and C tails in the PS-occluded E2∼P state. This intriguing study (37) with bile acids implies that flippase activities may be diversely regulated depending on the cellular context.

In human ATP8B1 heterologously expressed and purified from yeast cells, two conformations were observed in the autoinhibited E2P state: an open conformation, and a closed conformation in which the lipid entry site is unavailable for the E2P state as discussed above (39). Activation of this ATP8B1 required the removal of both N and C tails and the presence of phosphoinositides, in particular PI(3,4,5)P_3_ (phosphatidylinositol (3,4,5)-trisphosphate) (39). PI(3,4,5)P_3_ is a high affinity activator and consistently binds at a positively charged groove between M7 and M10 at the inner leaflet in both autoinhibited and activated E2P structures, reminiscent of PI4P bound at an equivalent site in the activated Drs2. However, unlike Drs2, the equivalent “helix switch” in ATP8B1 does not rotate upon activation, nor does it contain positively charged residues to bind PI(3,4,5)P_3_ (Fig. 4*C*). Consistent with this, truncation of the entire C-tail (ΔE1174-S1251) of ATP8B1, including removal of the “helix switch”, does not abrogate the requirement of phosphoinositide for activation. Moreover, the PI(3,4,5)P_3_ binding site is essentially unchanged for all observed conformations, suggesting that phosphoinositides could potentially bind at any state during the catalytic cycle (39). Therefore, the mechanism of ATP8B1 activation by PI(3,4,5)P_3_ appears to be different from that of Drs2 activation by PI4P and remains to be established. Remarkably, the tail-cleaved (both N-tail and C-tail) and phosphoinositide-activated ATP8B1 (termed “phosphoinositide-activated ATP8B1” hereafter) also exhibited a very different lipid cargo profile. Unlike the cargo-activated ATP8B1 that strongly prefers PS over PE and PC (37), the phosphoinositide-activated ATP8B1 prefers PE and PC over PS, both of which carry a smaller headgroup (39). The physiological relevance of these observations requires further investigation.

Protein phosphorylation adds another layer of regulation for the phosphoinositide-activated ATP8B1. An exogenous synthetic C-tail peptide (Arg-1205 to Ser-1251), long enough to span the cytosolic domain interfaces, efficiently inhibited the ATPase activity of activated ATP8B1. However, phosphorylation of Ser-1223 located on the short helix of the C-tail peptide was found to reduce its ability to inhibit the activated enzyme and increase the IC_50_ by over 17-fold (Fig. 4*A*) (38). These data signify the role of protein phosphorylation in regulation of ATP8B1. Interestingly, the yeast Drs2 is not subject to inhibition by the C-tail peptide derived from ATP8B1, suggesting that the C-tail-mediated inhibition is protein-specific. Both tail cleavage and phosphoinositide binding are found to be essential for ATP8B1 activation, however, regulation by protein phosphorylation could be abolished if proteolytic cleavage of the C-tail occurs *in vivo*. Moreover, the flippase would be irreversibly activated if proteolytic cleavage of the tails does occur *in vivo*. Alternatively, the N and C tails could be sequestered by unidentified binding partners, as suggested for the yeast Drs2 (27, 28). Additionally, phosphorylation of Ser-1223 at the short helix makes the C-tail less inhibitory, although it is unclear if the serine phosphorylation alone could fully activate the flippase without cleavage of inhibitory tails.

## Lipid cargo caught in the act: What and how do flippases flip?

Visualizing the transition from the lipid-loaded E2P state to the lipid-occluded E2∼P state sheds light on both transport mechanisms and lipid cargo specificity. The closing of the lipid entry by M1 and M2 in the E2∼P state is induced by a 25° rotation of the A-domain toward the P-domain in ATP11C (31). The A-domain rotates similarly by 20° in the phosphoinositide-activated ATP8B1 (Fig. 3*E*) (39). Interestingly, in the phosphoinositide-activated ATP8B1’s E2∼P state, the N-domain tilts toward the A-domain by 16° transitioning from the E2P state (Fig. 3*F*), while remaining fully dissociated from the P-domain. The N-domain tilts similarly during this transition in both ATP8A1 and Drs2 as well (26, 72). Presumably, the N-domain tilt promotes A-domain rotation thereby facilitating the dephosphorylation occurring at the interface between the A and P domains. The A-domain rotation in the E2∼P state reorients the conserved glutamate in the DGET motif to abstract a proton from a water molecule for in-line nucleophilic attack on the aspartyl phosphate moiety in the P-domain (48). However, structures have been reported in which the assigned E2∼P state resembles the E2P state without the aforementioned A and N domain movements, such as in Dnf2, Neo1, and to a lesser extent, the lipid cargo-activated ATP8B1 (32, 34, 37). Because lipid occlusion is a signature of the E2∼P state, these observations may suggest that binding of both lipid cargo and the phosphate analog AlF_4_^−^ is required to induce the transition. Indeed, in the reported structures of Dnf2 and Neo1, no occluded lipid was captured in their assigned respective E2∼P state (32, 34). The assigned E2∼P state structure of the lipid cargo-activated ATP8B1 may be an intermediate between the E2P and E2∼P state, given that the A-domain rotates only halfway through, and the lipid cargo is not fully occluded. This exemplifies another difference between phosphoinositide-activated and lipid cargo-activated ATP8B1.

**Figure 5:**
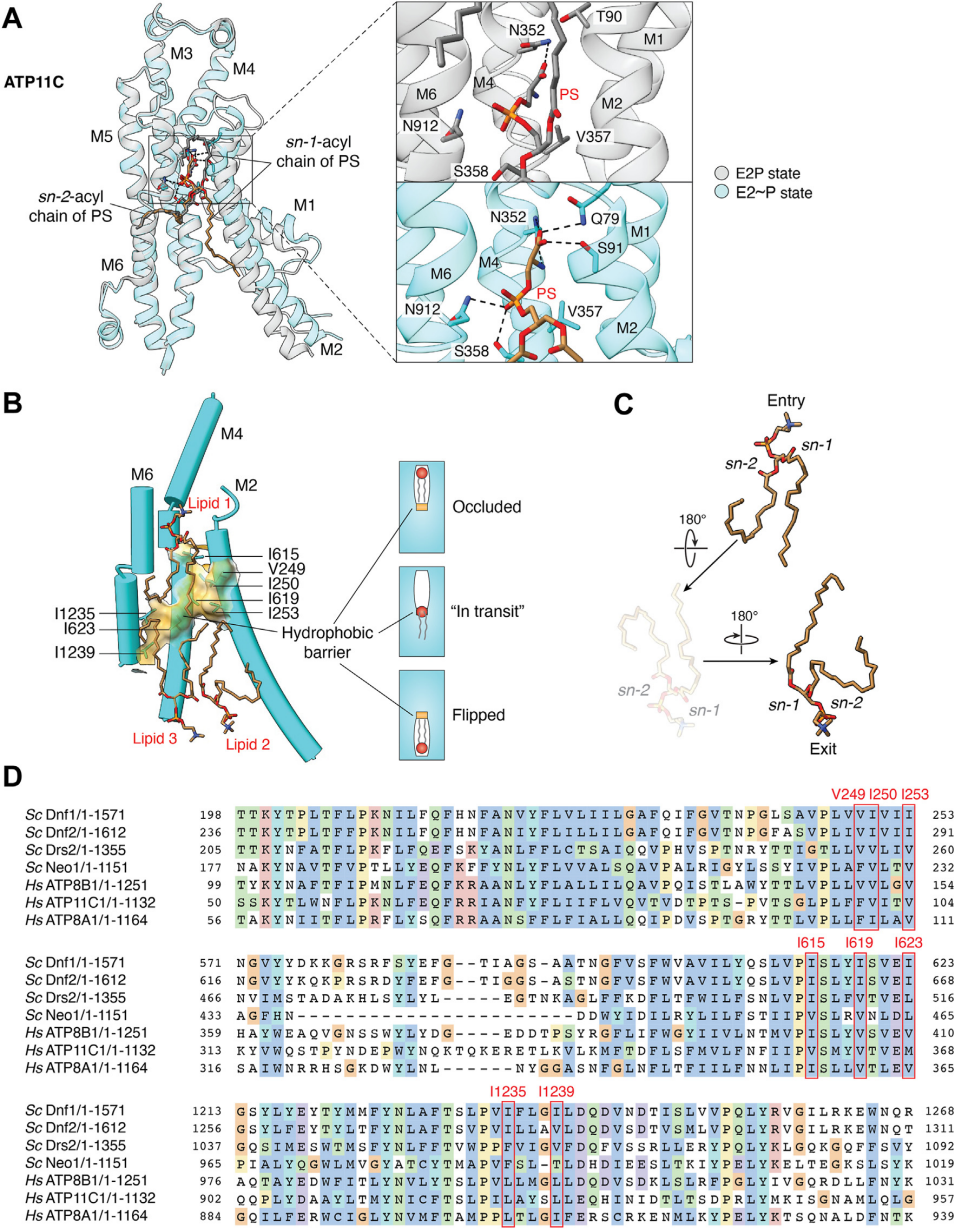
**Cargo lipid binding and occlusion (*A*) and the hydrophobic barrier model (*B*, *C* and *D*).***A*, comparison of lipid binding in the E2P state (PDB 7BSU) with bound PS shown as *gray sticks* and lipid occlusion in the E2∼P state (PDB 7BSV) with occluded PS in *brown sticks*. The “hydrophobic gate” residue and key residues interacting with the lipid headgroup, phosphate, and backbone are shown as gray sticks for the E2P state and cyan sticks for the E2∼P state. The right panels are enlarged views of the area in the square in the left panel, showing a side-by-side comparison of detailed interactions with the lipid for the two states. *B*, the invariant hydrophobic barrier in the middle of the putative lipid transport pathway is shown in transparent hydrophobic surface (PDB 7KYC). The conserved hydrophobic residues are shown in cyan sticks. Lipids are shown in brown sticks, with Lipid 1 in the lipid entry site, Lipid 2 in the putative lipid exit site, and Lipid 3 adjacent to Lipid 2. The right panel is a schematic illustration of the lipid flipping process, with the hydrophobic barrier shown as a small gold band. *C*, structures of two cargo lipids, one before and one after flipping (PDB 7KYC). The two PC lipids found in the putative transport pathway are shown as *brown sticks*. Another flipped PC lipid based on the PC at the lipid entry in the inner leaflet is shown in a dimmed representation to indicate a possible two-step flipping process, as marked by the *arrows*. *D*, sequence alignment of the flippase hydrophobic barrier regions. Hs, *Homo sapiens*; Sc, *Saccharomyces cerevisiae*. Note that the numbering of ATP11C isoform 1 (UniProtKB Q8NB49-1) used for the alignment is +3 higher than that of the ATP11C cryo-EM structures (NCBI Reference Sequence XM_005262405.1); ATP8A1 isoform 1 (UniProtKB Q9Y2Q0-1) is used for the alignment while the ATP8A1 cryo-EM structures have the sequence of isoform 2 (UniProtKB Q9Y2Q0-2).

Transitioning from the E2P state to the E2∼P state, the lipid headgroup moves slightly deeper and forms more hydrogen bonds in the cargo groove, and the lipid *sn-1*-acyl chain is better stabilized *via* hydrophobic interactions with M2 and M4 (Fig. 5*A*). Several determinants have been suggested for lipid cargo recognition, including headgroup noncovalent interactions, glycerol backbone stereochemistry, acyl chain length, condensation chemistry at the lipid *sn-2* position, and also the water network in which the lipid headgroup forms multiple water-mediated interactions within the cargo transporting groove (26, 31, 39, 73). Among all solved E2∼P state structures containing an occluded lipid cargo (Fig. 5*A*), a conserved asparagine residue in M6 (N1050 in Drs2, N882 in ATP8A1, N989 in ATP8B1, and N912 in ATP11C) H-bonds with the lipid phosphate moiety, which could be further stabilized by an invariant serine in the M4 PISL motif when the serine is in the right rotamer status. This serine can also take on an alternative rotamer to interact with the carbonyl of the lipid *sn-2* ester, and the presence of this ester moiety at the *sn-2* position was proposed to be a key determinant for cargo recognition in ATP8B1 (39). Interestingly, the murine ATP8A1 exclusively recognizes the naturally occurring *sn*-1,2-glycerol backbone of PS, and PS with acyl chains shorter than 10 carbon atoms in length only weakly stimulated the ATPase activity of the human ATP8A1 (26, 73).

One would assume that flippases recognize different lipids based primarily on their individual headgroups. However, accumulating structural evidence has revealed a very similar lipid cargo binding mode, regardless of the identity of the headgroup (Fig. 5*A*). In the E2P state, the lipid headgroup is anchored by a largely conserved asparagine in M4 (S503 in Drs2, N352 in ATP8A1 and ATP11C, and N397 in ATP8B1) and a conserved threonine in M2 (T249 in Drs2, T100 in ATP8A1, T143 in ATP8B1 and T90 in ATP11C). In the E2∼P state, new interactions form between a conserved glutamine in M1 (Q237 in Drs2, Q88 in ATP8A1, Q131 in ATP8B1 and Q79 in ATP11C) and the lipid headgroup, leading to the closure of the lipid entry site. Moreover, the lipid entry closure breaks the headgroup interaction with the M2 threonine and forms a new interaction with an adjacent threonine (T250 in Drs2, T101 in ATP8A1, T144 in ATP8B1 and S91 in ATP11C). Indeed, despite their distinct headgroups, PC, PS and PI (phosphatidylinositol) could all be occluded and stimulate the ATP8B1 ATPase activity, to varying degrees. This apparent “non-specificity” was attributed to the wide lipid entry site in its E2P state, as well as the formation of distinct water networks in the lipid groove upon interaction with M1 and M2 to facilitate dephosphorylation in the E2∼P state (39). Presumably, a non-cargo lipid may also enter in the lipid entry site and bind in the E2P state provided that the headgroup fits into the opened groove. However, non-cargo lipids would fail to induce the above-described conformational changes required for transitioning into the E2∼P state, mediated by M1 and M2, and the water network around the headgroup. In support of this hypothesis, PC is not a cargo of ATP11C, yet it inhibits PS-dependent ATPase activity in a competitive manner, with an apparent binding affinity similar to that of PS (31). Taken together, the lipid cargo specificity may be determined less by its binding mode but more by its ability to trigger the conformational change that leads to lipid occlusion.

How exactly the lipid flipping occurs remains to be established as no lipid cargo “in transit” has yet been captured. Among the three proposed models depicting lipid transport, namely, the two-gate model, the central cavity model, and the hydrophobic gate model, the hydrophobic gate model explains how the lipid cargo is flipped, which is also in good agreement with accumulating structural evidence (53, 54, 74). Indeed, all solved flippase structures unequivocally reveal an invariant “hydrophobic barrier” in the middle of the putative lipid transport pathway. The barrier also appears to be more extensive than the previously proposed gate, including not only the aforementioned isoleucine residue of the PISL motif but also other conserved hydrophobic residues in M2, M4, and M6 (Fig. 5, *B* and *D*). Together, these residues constitute a hydrophobic barrier that presumably the polar headgroup of lipid cargo has to cross. Interestingly, this barrier is observed in all flippase structures elucidated so far regardless of the conformational state, and on each side of the barrier, the two hydrophilic grooves presumably accommodate the pre-flip and post-flip lipid headgroup, respectively. A heroic kinetic analysis of 130 mutants of ATP8A2’s ATPase activity suggests that the headgroup “sliding” of lipid cargo in each hydrophilic groove is analogous to unzipping a “zipper” of salt bridges and hydrogen bonds between M2 and M4 (75).

## Flippase in the lipid bilayer: is the membrane thickening or thinning?

Structures in states post the E2∼P state are not yet available. However, bound lipids have been observed at or around the putative lipid exit site in some structures in the E2P state as discussed above. Curiously, if a *bona fide* flipped lipid cargo also assumes the same binding mode, this would imply that the lipid cargo has also rotated 180° around the vertical axis during transport, in addition to the anticipated flipping *via* a 180° rotation around the horizontal axis (Fig. 5*C*). Alternatively, this unexpected additional rotation could also happen during cargo release into the inner leaflet. The *sn-1*-acyl chain of these bound lipids is also stabilized between M2 and M4, similar to that of the occluded lipids at the lipid entry site. Interestingly, these lipids have moved partially out of the inner leaflet region, appearing out of register and about 10 Å into the cytoplasmic space relative to the inner leaflet lipids (32, 36). Consistent with the apparent membrane thickening, a distended inner leaflet was also observed in the nanodisc-reconstituted ATP11C, in which the conserved Arg-113 (Arg-265 in Dnf1) in M2 “snorkels” toward the negatively charged lipid headgroups. This membrane perturbation likely facilitates the lipid cargo release into the inner leaflet (35).

**Figure 6:**
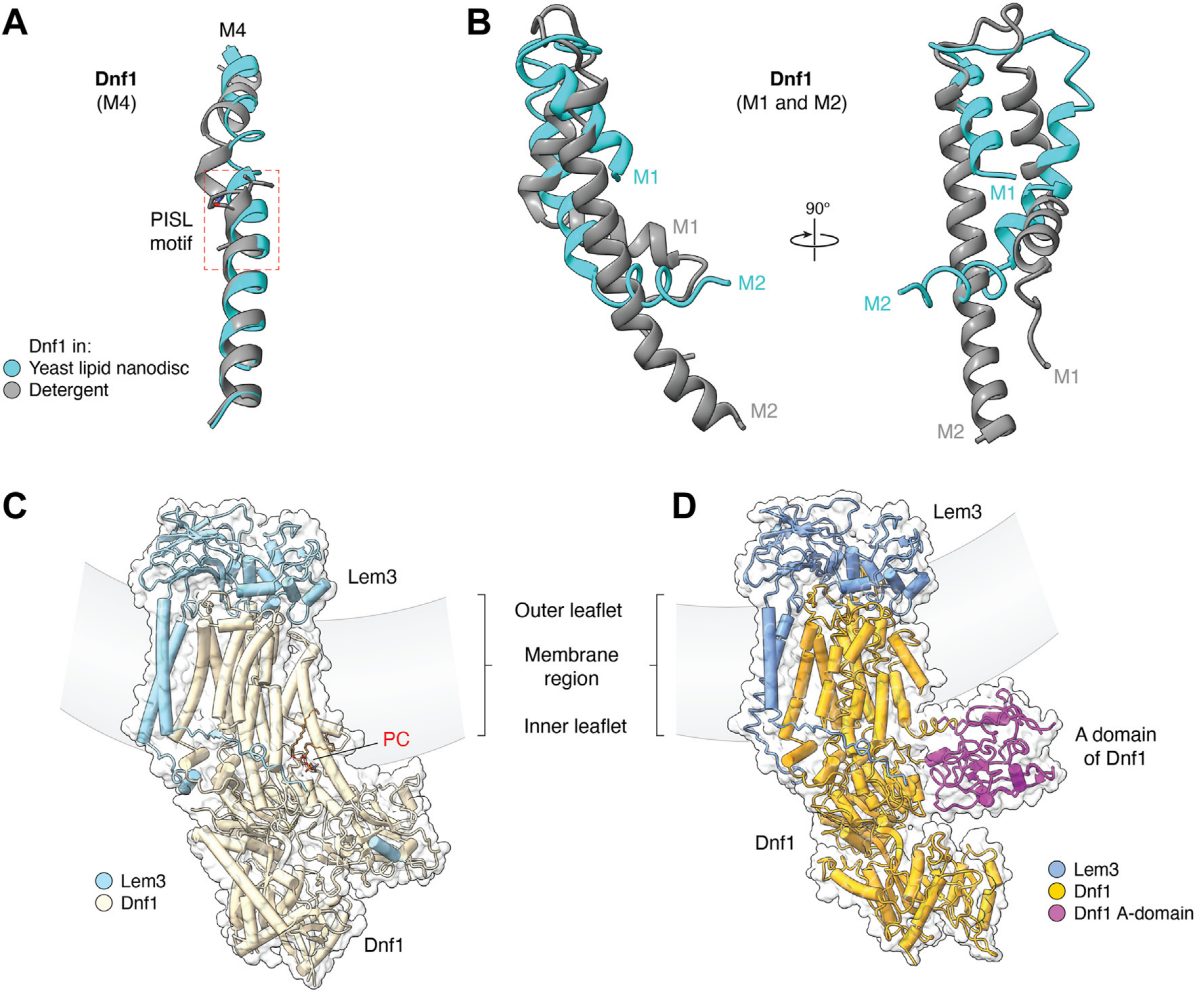
**Unkinked M4 (*A*) and rearranged M1 and M2 (*B*) in the orphan state of Dnf1 in yeast lipid nanodiscs and the proposed membrane curved around Dnf1 bound with PC at the putative exit (*C*) and with an over-rotated A-domain (*D*).***A* and *B*, structural comparisons between the presumed E2P state of Dnf1 in yeast lipid nanodiscs (PDB 7F7F) and the typical E2P state of Dnf1 in detergents (PDB 7WHV). The PISL motif in M4 is boxed in red *dashed lines*. *C*, cartoon representation of Dnf1–Lem3 in detergents (PDB 7KYC) with transparent surface and a curved *gray* band indicating the membrane region. The bound PC is shown as *brown sticks*. *D*, cartoon representation of Dnf1–Lem3 in yeast lipid nanodiscs (PDB 7F7F) with transparent surface and a curved *gray* band indicating the membrane region.

On the other hand, an apparent membrane thinning effect has also been described for flippases reconstituted in nanodiscs, with a highly mobile A-domain dipping into the inner leaflet of the lipid bilayer (29, 36). Remarkably, the two structures solved for the yeast Dnf1 reconstituted in nanodiscs with yeast lipids represent rare “orphan” states, despite the use of the nonhydrolyzable ATP analog AMPPCP and the phosphate analog BeF_3_^−^, respectively. Three unique features stand out as they are only shared by these two orphan state structures (Fig. 6, *A* and *B*): 1) M4 has lost its signature kink harboring the PISL motif and become a straight α-helix, leading to a disrupted lipid entry groove; 2) the parallel M1 and M2 have dramatically rearranged to become crossed, further compromising the lipid binding; 3) the A-domain has apparently over-rotated into the inner leaflet, perhaps enabled by a positively charged patch on this domain. However, the *C*. *thermophilum* Dnf1 lacks such a positively charged patch yet its A-domain has still over-rotated into the inner leaflet (29), indicating that an electrostatic interaction of the A-domain with the inner leaflet lipids is not essential. Coincidentally, the distance the A-domain protrudes upward into the membrane (10 Å) is similar to the distance the bound lipids in the putative lipid exit site move downward into the cytosol (10 Å). Taken together, the apparent contradictory membrane thickening *versus* thinning effect can be reconciled by the membrane bending around the flippases (Fig. 6, *C* and *D*). Indeed, the flippase activity can expand the inner leaflet at the expense of the outer leaflet, leading to a buildup of mechanical strain that has to be relieved by deformation—forming curved membranes (76, 77). A curved membrane may facilitate the release of flipped lipids into the inner leaflet and accommodate an over-rotated A-domain toward the membrane.

The biological significance of the orphan states of the yeast Dnf1 remains to be elucidated. However, their existence suggests that membrane lipid composition can influence the protein conformation and regulate the flippase activity. It is worth noting that, unlike its mammalian counterpart, PC is predominantly found in the inner leaflet of the yeast plasma membrane instead of the outer leaflet (78). The intriguing observation that Dnf1 in a 10% PC and 90% PS lipid bilayer remained active but became catalytically incompetent in a lipid bilayer with 31% PC, 8% PS, and other lipids could indicate new mechanisms for flippase inhibition.

## Future perspectives

Lipid flippases have come a long way, and our mechanistic understanding of this lipid transporter has been substantially improved in the past several years by the high-resolution structures of different flippases captured in various conformational states along the catalytic cycle. Despite the abundance of solved structures, the molecular mechanisms of lipid transport remain incompletely understood. There is a general lack of structural snapshots after the lipid-occlusion state, which may be a major challenge for future structural efforts. Given the physiological significance of membrane deformation by flippases, additional insights will likely be obtained by studying flippases in a membrane setting, such as in nanodiscs, proteoliposomes, or even cell-derived vesicles that preserve asymmetric native membranes (79). While the conventional description of the flippases in the E1/E2 scheme will likely continue to be used, we caution that E1 and E2 do not necessarily define the facing orientation (*i.e.*, inward-facing or outward-facing) as demonstrated in Ca^2+^-ATPases (51, 80). Although most P-type ATPase structures have been assigned to the individual states based on the Post-Albers scheme, the E1/E2 model has shortcomings that need to be adapted if not abandoned (81, 82, 83).

## Conflict of interest

The authors declare that they have no conflicts of interest with the contents of this article.
